# MSAIndelFR: a scheme for multiple protein sequence alignment using information on indel flanking regions

**DOI:** 10.1186/s12859-015-0826-3

**Published:** 2015-11-23

**Authors:** Mufleh Al-Shatnawi, M. Omair Ahmad, M. N. S. Swamy

**Affiliations:** Department of Electrical and Computer Engineering, Concordia University, 1455 De Maisonneuve Blvd. W., Montreal, H3G 1M8 Quebec Canada

**Keywords:** Multiple sequence alignment, Indel flanking regions, PPM IndelFR predictor, Dynamic programming

## Abstract

**Background:**

The alignment of multiple protein sequences is one of the most commonly performed tasks in bioinformatics. In spite of considerable research and efforts that have been recently deployed for improving the performance of multiple sequence alignment (MSA) algorithms, finding a highly accurate alignment between multiple protein sequences is still a challenging problem.

**Results:**

We propose a novel and efficient algorithm called, MSAIndelFR, for multiple sequence alignment using the information on the predicted locations of IndelFRs and the computed average log–loss values obtained from IndelFR predictors, each of which is designed for a different protein fold. We demonstrate that the introduction of a new variable gap penalty function based on the predicted locations of the IndelFRs and the computed average log–loss values into the proposed algorithm substantially improves the protein alignment accuracy. This is illustrated by evaluating the performance of the algorithm in aligning sequences belonging to the protein folds for which the IndelFR predictors already exist and by using the reference alignments of the four popular benchmarks, BAliBASE 3.0, OXBENCH, PREFAB 4.0, and SABRE (SABmark 1.65).

**Conclusions:**

We have proposed a novel and efficient algorithm, the MSAIndelFR algorithm, for multiple protein sequence alignment incorporating a new variable gap penalty function. It is shown that the performance of the proposed algorithm is superior to that of the most–widely used alignment algorithms, Clustal W2, Clustal Omega, Kalign2, MSAProbs, MAFFT, MUSCLE, ProbCons and Probalign, in terms of both the *sum–of–pairs* and *total column* metrics.

**Electronic supplementary material:**

The online version of this article (doi:10.1186/s12859-015-0826-3) contains supplementary material, which is available to authorized users.

## Background

Alignment of multiple protein sequences is a crucial step in bioinformatics analyses, and is used in many applications including sequence annotation, phylogenetic tree estimation, evolutionary analysis, secondary structure prediction and protein database search [1, 2]. Multiple sequence alignment (MSA) allows us to identify parts of the protein sequences that are similar to one another with gaps (spaces) inserted in such a way that similar parts of these sequences can be easily identified [3]. The concept of a gap in an alignment is important, since the gap locations indicate the locations of insertion or deletion (indel) mutation events in protein sequences. It should be noted that the insertion or deletion of an entire subsequence often occurs as a single mutational event, and such single mutational events can create gaps of varying sizes [4]. In recent years, considerable effort has been devoted to the development of MSA algorithms that can efficiently detect mutations and generate highly accurate alignments. Some of the significant algorithms are Clustal W2 [5], Clustal Omega [6], Kalign2 [7], MSAProbs [8], MAFFT [9, 10], MUSCLE [11], ProbCons [12] and Probalign [13].

Clustal W2, Clustal Omega, Kalign2 and MSAProbs are *progressive alignment* algorithms, while MAFFT, MUSCLE, ProbCons and Probalign generate an initial alignment using the progressive alignment algorithm and then iteratively refine this alignment to achieve higher alignment accuracy. A progressive alignment algorithm involves three steps: (i) calculations of the pairwise distances between all pairs of sequences to determine the similarity of each pair of sequences, (ii) construction of a guide tree based on the distance matrix, and (iii) finally, alignment of the sequences according to an order determined by the guide tree [4, 14].

Clustal W2 and Clustal Omega are derived from Clustal W [15]. Clustal W2 calculates the pairwise distances between all pairs of sequences using the k–tuple method [16], and then constructs the guide tree using the *unweighted pair group method with arithmetic mean* (UPGMA) [17]. Clustal Omega is the latest MSA algorithm in the Clustal family, and the main improvements of Clustal Omega over Clustal W2 are as follows: (i) it can align any number of protein sequences, (ii) it allows the use of a profile hidden Markov model, derived from an alignment of protein sequences related to the input sequences, and (iii) it allows the user to choose the number of iterations, in the absence of which it is a progressive algorithm by default. Further, Clustal Omega is the most accurate and scalable MSA algorithm amongst the Clustal family. In Kalign2, the pairwise distances between all pairs of sequences are estimated based on the the Muth–Manber string matching algorithm [18] and the guide tree constructed using UPGMA. MSAProbs [8] is based on combining a pair hidden Markov model with partition functions to calculate the posterior probabilities, which are used in estimating the pairwise distance matrix. In MSAProbs, the guide tree constructed using UPGMA. It should be noted that MSAProbs is currently the most accurate alignment algorithm. The alignment algorithms MAFFT, MUSCLE, ProbCons and Probalign are not fully progressive. In these algorithms, iterative refinement is performed to improve the alignment and the guide tree constructed using UPGMA for the next iteration.

Multiple sequence alignment algorithms use an *objective function* (OF) to measure the quality of an alignment. A simple OF should include a gap penalty function to score the gaps and substitution matrices to measure the similarity of amino acid pairs. The most widely used gap penalty function is the *affine gap penalty* (AGP), given by *g*(*k*)=*g*_*o*_+*k* *g*_*e*_ for a gap of length *k*. The function *g*(*k*) involves two parameters, *g*_*o*_ and *g*_*e*_, *g*_*o*_ representing a gap opening penalty at a specific position in the protein sequence and *g*_*e*_ representing an extension penalty for extending the gap. This linear AGP function has the advantage of simplicity and ease of use in MSA algorithms. However, this penalty function is restrictive in the sense that the two parameters remain fixed for aligning different positions in the protein sequence.

MSAProbs, Kalign2, ProbCons and Probalign are MSA algorithms for which an AGP function is used. In MSAprobs, ProbCons and Probalign, fixed parameters are used for the AGP function, wherein a gap opening penalty of −22 and a gap extension penalty of −1 are used by default [8, 12, 13]. Kalign2 determines the default gap penalties for protein alignments by training on a BAliBASE 3.0 benchmark [19] in order to obtain optimal alignment results. In the MAFFT, MUSCLE, Clustal W2 and Clustal Omega MSA algorithms, a gap opening penalty (GPO) and a gap extension penalty (GPE) values are initially specified; then, these algorithms automatically attempt to choose appropriate gap penalties according to some specific rules. The algorithms MAFFT and MUSCLE use an AGP function, wherein the default values are modified depending on the number of existing gaps at a particular position for a given profile [10, 11]. Clustal W2 and Clustal Omega use an AGP function, wherein a gap opening penalty (GPO) and a gap extension penalty (GPE) are initially set by the user from a menu, and then, these algorithms automatically attempt to choose appropriate gap penalties for each sequence alignment according to the features of the input sequences, such as sequence divergence, length, and local hydrophobic amino acids. It should be noted that the choice of the AGP parameters has a substantial effect on the alignment accuracy [2, 20, 21], and the widely–used AGP works well for closely related or similar sequences, but they are less effective for highly diverged or dissimilar sequences. As a consequence, there has been a growing interest in conducting multiple sequence alignment with more general and flexible gap penalty functions.

In the present work, we propose a novel and efficient algorithm for multiple sequence alignment, referred to as MSAIndelFR, that incorporates the information concerning the predicted indel flanking regions (IndelFRs). The key innovation in MSAIndelFR is the use of the predicted information about IndelFRs to propose a new *variable gap penalty* (VGP) function, wherein the gap opening penalty is position–specific and the gap extension penalty is region–specific. It should be noted that the predicted IndelFRs are the most likely regions for the gaps to be introduced in the protein sequence alignment, since they are strongly related to indel mutations [22–26]. Therefore, it is expected that more accurate alignments can be obtained by integrating the predicted information about IndelFRs into the gap penalty function. To the best of our knowledge, using the predicted information about IndelFRs in multiple sequence alignment is novel. The performance evaluation results on MSAIndelFR indeed confirm that incorporating the predicted information about the indel flanking regions improves the alignment accuracy.

## Methods

### Indel flanking regions (IndelFRs)

**Fig. 1:**
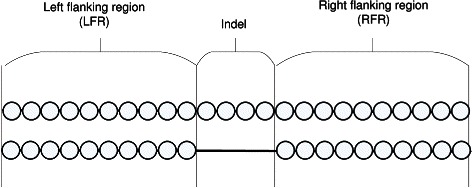
The indel and the flanking regions

When a pair of protein sequences is aligned, a gap in any of the two sequences is defined as an *indel region*. Segments of these two sequences immediately before and after an indel region are called *flanking regions*, as shown in Fig. 1. In [27], an indel along with its left and right flanking regions is referred to as an *indel flanking region (IndelFR)*. The results in [27] strongly suggest that the IndelFRs for a given protein sequence are confined only to the IndelFR segments, which are the segments of the protein sequence to which all the predicted IndelFRs collectively belong to.

### PPM IndelFR Predictor

A technique for building the IndelFR predictor for a given protein fold, based on the *prediction by partial match* (PPM) [28], was proposed in [27]. This PPM IndelFR predictor for a given protein fold contains two variable–order Markov models, one for predicting the left flanking and the other for predicting the right flanking regions. It is has been shown in [27] that the best choice for the value of *D*, the memory length of the PPM IndelFR predictor, is 4.

Given a test protein sequence **S** ^*n*^=*s*_1_ *s*_2_ *s*_3_⋯*s*_*n*_ of length *n*, the PPM IndelFR predictor scans it using a running window of length *L*=10 moving it one amino acid at a time, to determine whether the string of amino acids within a window contains an IndelFR or not. It should be noted that the impact of an indel on its flanking regions reduces dramatically as we move away from the indel, and is negligible after 10 amino acids [23].

**Fig. 2:**
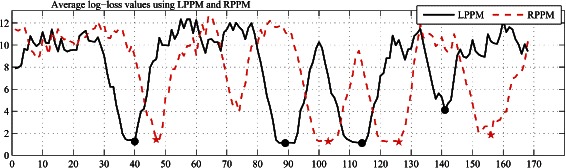
The left and right average log-loss values for the *d1liab_* using left PPM (LPPM) and right PPM (RPPM) IndelFR predictor. The solid dots represent the start locations of the predicted left flanking regions and the stars that of the predicted right flanking regions

The PPM IndelFR predictor, with *D*=4, computes the left and right average values for each position in the protein sequence, and then uses the algorithm in [27] to extract the predicted locations of IndelFRs in the protein sequence. In [27], the average log–loss value for window of length *L*=10 at position *i*, **win**_*i*_=*s*_*i*_ *s*_*i*+1_⋯*s*_*i*+9_, in the sequence is defined as

(1) $$ \begin{aligned} logloss & P(\mathbf{win}_{i})=\\ &-\frac{1}{L}\left(\log P_{0}(s_{i}) + \log P_{1}(s_{i+1}|s_{i})+ \right.\\ &\left. \log P_{2}(s_{i+2}|s_{1}s_{i+1})+\cdots+ \right.\\ & \left.\log P_{D}(s_{i+L-1}|s_{i+L-1-D}\cdots s_{i+L-2})\right)  \end{aligned}  $$

where the logarithm is taken to base 2. For the purpose of illustration, the left and right average log–loss values for the protein sequence *d1liab_* at different positions are shown in Fig. 2.

The PPM IndelFR predictors for 11, 14 and 18 protein folds from different protein classes: *All–* *α* *proteins*, *All–* *β* *proteins* and *α* and *β* proteins (a/b), respectively, have been constructed in [27] and for convenience, included in the supplementary data of this paper (Additional file 1: Tables S1–S3). Hence, we have 43 different PPM IndelFR predictors. It should be noted that the PPM IndelFR predictors were trained using the IndelFR database [22], which in turn provided IndelFRs for some selected protein sequences belonging to certain selected protein folds from the SCOP database [29]. Moreover, it should be pointed out that the PPM IndelFR predictors in [27] do not use directly any protein structure information (alpha, beta or coil) and use only the information about the positions, lengths, and amino acid compositions of the indel flanking regions listed in the IndelFR database; however, the IndelFR database itself has used the structure-based sequence alignment to extract the information concerning the indel flanking regions. In [27], it has been demonstrated that once the PPM IndelFR predictor is built for a given protein fold, it can be used to compute the average log–loss values for any protein sequence belonging to this protein fold. Hence, we will be able to compute the average log–loss values, and then use the algorithm in [27] to predict the IndelFRs for protein sequences that are available in the selected protein folds, even though the IndelFR database did not provide IndelFRs for these protein sequences.

### MSAIndelFR algorithm

**Fig. 3:**
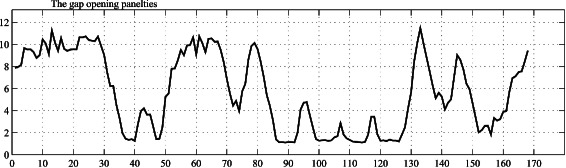
The gap opening penalties for the *d1liab_* extracted from the left and right average log–loss values shown in Fig. 2

In this section, we propose an algorithm for MSA, termed MSAIndelFR algorithm, that makes use of the computed average log–loss values and the predicted IndelFRs from the PPM IndelFR predictor. The results in [27] concerning PPM IndelFR predictor have shown that the computed average log–loss values in and around an IndelFR are much smaller than that in other regions. In view of this observation, we combine the left and right average log–loss values for any given protein sequence **S** ^*n*^=*s*_1_ *s*_2_ *s*_3_⋯*s*_*n*_ of length *n* to propose a *position–specific gap opening penalty* function. The proposed position–specific gap opening penalty at a particular position *i* in the sequence, *G* *P* *O*_*i*_, is given by

(2) $$ \begin{aligned} & GPO_{i}=\\& \left\{ \begin{array}{ll} \text{min}(LPPM_{i}, RPPM_{i}), & 1\leq i\leq(n-L+1)\\ GPO_{(n-L+1)}, & (n-L+1)< i \leq n \end{array}\right.  \end{aligned}  $$

where *L* *P* *P* *M*_*i*_ and *R* *P* *P* *M*_*i*_ are, respectively, the left and right average log–loss values at position *i*. It is seen from this equation that *G* *P* *O*_*i*_, for (*n*−*L*+1)<*i*≤*n*, is chosen to be equal to the gap opening penalty at position *i*=*n*−*L*+1. The gap opening penalties at different positions for *d1liab_* are shown in Fig. 3.

In addition to using the gap opening penalty function *G* *P* *O*_*i*_, we use the predicted IndelFRs to propose a *region–specific gap extension penalty* function. As mentioned in the introduction, the predicted IndelFRs are the most likely regions for the gaps to be introduced in the protein sequence, since they are strongly related to indel mutations [22–26]. Moreover, a single indel mutation event often affects several adjacent amino acids in a protein sequence [4]. This is taken into consideration in the proposed definition of the gap extension penalty at position *i* in the protein sequence, *G* *P* *E*_*i*_ :

(3) $$ GPE_{i}=\left\{ \begin{array}{ll} 0, & \text{if position} \; i \in IndelFRs \\ GPO_{i}, &\text{otherwise}\; \end{array}\right.   $$

In the other words, a zero value is assigned to *G* *P* *E*_*i*_, if the gap introduced at position *i* is in an IndelFR, while it is equal to *G* *P* *O*_*i*_ if *i* is not in an IndelFR.

As explained above, the gap penalty functions are set using the IndelFRs predicted by the PPM IndelFR predictor. However, the predictor for a given protein fold is not trained using any benchmark or any of its subsets. In fact, it is trained using the IndelFR database [22].

### New FASTA format

We modify the standard FASTA format to include information about the position–specific gap opening penalty and the predicted locations of IndelFRs into the standard FASTA format (Additional file 1: Section 1). Hence, the input protein sequences to the proposed MSAIndelFR algorithm should be written using the modified version of FASTA format, where the position–specific gap opening penalty and the predicted locations of IndelFRs are added after the main list of amino acids of the protein sequence.

### Alignment strategy

The alignment strategy is based on the standard progressive alignment method for aligning multiple protein sequences [14]. First, pairwise distances between input sequences are calculated to form a distance matrix. An accurate calculation of pairwise distances can be accomplished by performing all the pairwise alignments amongst the input sequences; however, this is not practical in view of time complexity, especially when the number of sequences is large, since any pairwise alignment requires quadratic time for completion [30]. Therefore, some of the existing MSA algorithms have used the k–tuple method [16] to calculate the pairwise distances approximately. It has been shown in [7] that the Muth–Manber string matching algorithm proposed in [18] to calculate the pairwise distances is more accurate than the k–tuple method; this algorithm finds the distance between two sequences by matching patterns that contain at most one error. For example, consider two sequences *ABCABCABC* and *ABDABDABD* that are 67 *%* identical. The k–tuple method (with a pattern length of 3) reports that these two sequences are not identical (i.e., share no exact patterns), while the Muth–Manber algorithm reports that these two sequences are 67 *%* identical. In view of this, we employ the Muth–Manber algorithm in our article to calculate the pairwise distances between the input protein sequences.

Since protein sequences are normally searched with short length patterns [7, 11, 15, 31], we search with patterns of length 3 of amino acids to calculate the pairwise distances. Then, a guide tree is constructed from the distance matrix using the unweighted pair group method with arithmetic mean (UPGMA) [17], which is the most popular method for guide tree construction and used in many MSA algorithms as the default option. Finally, sequences or profiles are aligned according to the order prescribed by the guide tree. Hence, at each internal node of the guide tree, two sequences, or two profiles or one sequence and one profile are aligned. The process of aligning sequences/profiles continues until the highest level of the guide tree is reached. For this purpose, we use the *dynamic programming* (DP) approach along with the proposed gap penalty functions, namely, the position–specific gap opening penalty function and the region-specific gap extension penalty function to align sequences/profiles.

### Dynamic programming with variable gap penalty function

We assume that the input protein sequences are evolutionary related over their entire lengths. Therefore, a global alignment of the input sequences will be obtained using the DP approach. The optimal alignment in the DP approach is the alignment which has the highest score, where the score of an alignment is found by using a gap penalty function and the substitution matrix *S*. It should be noted that any alignment between protein sequences is intended to reflect the cost of mutational events needed to transform one sequence to the another [4, 30]. In this article, we use a VGP function, which has two subfunctions: the position–specific gap opening penalty function *G* *P* *O*_*i*_ and the region–specific gap extension penalty function *G* *P* *E*_*i*_.

Let **A** ^*n*^=*a*_1_ *a*_2_ *a*_3_⋯*a*_*n*_ and **B** ^*m*^=*b*_1_ *b*_2_ *b*_3_⋯*b*_*m*_ be two sequences of length *n* and *m*, respectively. The DP approach finds the optimal alignment between **A** and **B** by computing the optimal alignments between all prefixes of **A** and **B**. The amino acids in **A** and **B** are assigned to one of three possible states: aligned, gap in sequence **A**, or gap in sequence **B** during the alignment process. These states are represented by three matrices in the DP approach. Let **A** [ 1:*i*]=*a*_1_ *a*_2_⋯*a*_*i*_ be a prefix of sequence **A**, **B** [ 1:*j*]=*b*_1_ *b*_2_⋯*b*_*j*_ be a prefix of sequence **B**, *M*(*i,j*) be the optimal score for aligning **A** [ 1:*i*] and **B** [ 1:*j*] given that *a*_*i*_ is aligned to *b*_*j*_, *I*_*A*_(*i,j*) be the optimal score given that *a*_*i*_ is aligned to a gap, and *I*_*B*_(*i,j*) be the optimal score given that *b*_*j*_ is aligned to a gap, where 1≤*i*≤*n* and 1≤*j*≤*m*. The recursive equations to find the various elements in the state matrices *M*(*i,j*), *I*_*A*_(*i,j*), and *I*_*B*_(*i,j*) are given by

(4) $$ {\fontsize{9}{6}\begin{aligned} & M(i,j)=s(a_{i},b_{j})\, + \\ & \text{max}\left\{ \begin{array}{ll} M(i-1,j-1), & \begin{aligned} & \text{With}~ a_{i-1} ~\text{aligned to}~ b_{j-1},\\ & \text{, align}~a_{i}~\text{to}~b_{j} \end{aligned} \\ I_{A} (i,j), & \begin{aligned} & \text{End a gap in}~\textbf{A},\\ & \text{, align}~a_{i}~\text{to}~ b_{j} \end{aligned}\\ I_{B} (i,j), & \begin{aligned} & \text{End a gap in}~ \textbf{B}, \\ & \text{, align}~a_{i}~\text{to}~b_{j} \end{aligned} \end{array}\right.  \end{aligned}}  $$

(5) $$ {\fontsize{9}{6}\begin{aligned} & I_{A}(i,j)=\\ & \text{max}\left\{ \begin{array}{ll} M(i-1,j)-\left(GP{O^{A}_{i}}+GP{E^{A}_{i}}\right), & \begin{aligned} & \text{Open a new} \\ & \text{gap in}~\mathbf{A} \end{aligned}\\ I_{A} (i-1,j)-GP{E^{A}_{i}}, & \begin{aligned} & \text{Extend an old}\\ & \text{gap in}~\mathbf{A} \end{aligned} \end{array}\right.  \end{aligned}}  $$

(6) $$ {\fontsize{9}{6}\begin{aligned} & I_{B}(i,j)=\\ & \text{max}\left\{ \begin{array}{ll} M(i,j-1)-\left(GP{O^{B}_{j}}+GP{E^{B}_{j}}\right), & \begin{aligned} & \text{Open a new} \\ & \text{gap in}~\mathbf{B} \end{aligned} \\ I_{B} (i,j-1)-GP{E^{B}_{j}}, & \begin{aligned} & \text{Extend an old}\\ & \text{gap in}~\mathbf{B} \end{aligned} \end{array}\right.  \end{aligned}}  $$

with

(7) $$ {\fontsize{9}{6}\begin{aligned} & M(0,0)=0, M(0,j)=GP{O^{B}_{1}}+\sum^{m}_{j=1} GP{E^{B}_{j}},\\ & M(i,0)=GP{O^{A}_{1}}+\sum^{n}_{i=1} GP{E^{A}_{i}}\\ & I_{A}(0,j)=-\infty, I_{B}(i,0)= -\infty  \end{aligned}}  $$

where *s*(*a*_*i*_,*b*_*j*_) can be obtained directly from the substitution matrix *S*, $GP{O^{A}_{i}}$ and $GP{E^{A}_{i}}$ are, respectively, the gap opening and extension penalty functions for the sequence **A**, and $GP{O^{B}_{j}}$ and $GP{E^{B}_{j}}$ are the corresponding penalty functions for the sequence **B**. Once the computation of *M* is completed, it contains the maximum alignment score, and a trace back procedure is used to retrieve the alignment between **A** and **B**.

In this article, we implement the memory efficient DP algorithm proposed in [32], which can align two sequences of lengths, say *n* and *m* (*n*≥*m*), with a time complexity of *O*(*m* *n*) and a space complexity of *O*(*n*). Since it has been shown in [33] that the selection of a particular substitution matrix does not noticeably affect the alignment accuracy, and that there is little difference in the alignment accuracy using BLOSUM [34], PAM [35] or GONNET [36] as the substitution matrix, we use GONNET250 as the substitution matrix.

In order to continue aligning sequences/profiles until the highest level of the guide tree is reached, we need the gap penalty functions: *G* *P* *O*_*i*_ and *G* *P* *E*_*i*_, for each profile. For example, consider the alignment of two sequences, say, **A** and **B** at the lowest level of the tree to produce the profile **C**. The position–specific gap opening penalty function for profile **C** is defined to be

(8) $$ \begin{aligned} & GP{O^{C}_{i}}=\\ &\left\{ \begin{array}{ll} GP{O^{A}_{j}} + GP{O^{B}_{k}}, & \begin{aligned} &~~~\text{if}~ a_{j}~\text{is aligned with} \\ & ~~~b_{k}~\text{at position}~ i \end{aligned}\\ GP{O^{A}_{j}}, & \begin{aligned} & \text{~~~if there is a gap in}\\ & \mathbf{~~~B}~\text{at position}~i \end{aligned}\\ GP{O^{B}_{k}}, & \begin{aligned} & \text{~~~if there is a gap in}\\ & \mathbf{~~~A}~ \text{at position}~i \end{aligned} \end{array}\right.  \end{aligned}  $$

where $GP{O^{A}_{j}}$, $GP{O^{B}_{k}}$ and $GP{O^{C}_{i}}$ are the gap opening penalty functions at positions *j*, *k*, and *i* for **A**, **B** and **C**, respectively. In a similar manner, we define the gap extension penalty function for **C**. This makes a gap more likely to occur at a position, where a gap already exists. If there is no gap at a position *i* in **C**, then the gap opening penalty is increased by adding both $GP{O^{A}_{j}}$ and $GP{O^{B}_{k}}$ to avoid introducing gaps at the aligned positions.

As already mentioned, the internal nodes of the guide tree are visited in a bottom–up order, and for each visited node a pairwise alignment of sequences/profiles is computed using the DP approach along with the proposed VGP function. The MSA associated with the root node is the final alignment.

## Results and discussion

The performance of MSA algorithms are usually evaluated on alignment benchmarks containing reference alignments. In this article, we use four popular benchmarks, namely, BAliBASE 3.0 [19], OXBENCH [37], PREFAB 4.0 [11] and SABmark 1.65 [38] to evaluate the performance of the proposed MSAIndelFR algorithm as well as that of the eight most–widely used MSA algorithms, namely, Clustal W2 version 2.1, Clustal Omega version 1.2.0, MSAProbs version 0.9.7, Kalign2 version 2.04, MAFFT version 7.184, MUSCLE version 3.8.31., ProbCons version 1.12 and Probalign version 1.4. For MAFFT, *auto* option is used with the maximum iterative refinement (*maxiterate* option) set to 1000, while the default options are used for all the other algorithms, including the proposed MSAIndelFR.

In the present article, we select the reference alignments from the above four benchmarks that have protein sequences belonging to one of the 43 protein folds (Additional file 1: Tables S1–S3). We use the PPM IndelFR predictor to compute the average log–loss values, and then use the algorithm in [27] to predict the IndelFRs for protein sequences that are available in the alignment benchmarks, even though the IndelFR database does not contain IndelFRs for these protein sequences. We would like to emphasize that no training is needed in the proposed MSAIndelFR algorithm. Further, it does not make use of the protein information (alpha, beta or coil) as input. It makes use of the computed average log–loss values and the predicted IndelFRs obtained from the PPM IndelFR predictors proposed in [27]. It should be noted that the PPM IndelFR predictors do not use any of the above–mentioned four benchmarks for their training, and the training set for any of the PPM IndelFR predictors is virtually different from the test set of the proposed MSAIndelFR algorithm on all the four benchmarks (See Section 5 of the Additional file 1).

We use the measures, *sum-of-pairs* (SP) and *total columns* (TC) [20], which are the most commonly used metrics, to evaluate and compare the performance of the various MSA algorithms. The SP value is defined as the number of correctly aligned amino acid pairs found in the test alignment divided by the total number of aligned amino acid pairs in the *core blocks* of the reference alignment, where the core blocks of the reference alignment refer to the regions for which reliable alignments are known to exist. We use the BENCH database (Edgar, R.C., http://www.drive5.com/bench) to determine the core blocks in the selected benchmarks. It should be noted that the *quality*(Q) metric used in [11] is equivalent to SP. The TC value is defined as the number of correctly aligned columns found in the test alignment divided by the total number of aligned columns in the core blocks of the reference alignment, and hence, gives the proportion of the total alignment columns that is recovered in the test alignment. A value of 1.0 for TC indicates perfect agreement between the test and reference alignments. It should be noted that the TC value is equivalent to the SP value in the case of pairwise alignment (as in the PREFAB benchmark). We calculate the SP and TC values employing the QSCORE software available at the website [39].^1^ In order to determine if the improvements, achieved in terms of the SP and TC values, by the proposed MSAIndelFR algorithm are statistically significant, the Wilcoxon matched-pair signed-rank test [40] is used.

### Evaluation using BAliBASE 3.0

For evaluating multiple sequence alignment algorithms, BAliBASE [19] is the most widely used benchmark. This benchmark contains 3D structural–based alignments that are manually refined. Out of the 386 reference alignments in BAliBASE, there are 186 alignments that have protein sequences which belong to one or the other of the 43 selected protein folds.

The average SP and TC values of MSAIndelFR as well as those of the other algorithms using this benchmark as reference are given in Table 1. The results show that MSAIndelFR achieves the highest SP and TC values. Specifically, it provides an average SP value of 86.23 *%* representing an improvement of 6.02 *%*, 1.37 *%*, 4.12 *%*, 4.29 *%*, 6.17 *%*, 10.37 *%*, 0.39 *%* and 0.78 *%* over that of MSAProbs, MAFFT, MUSCLE, Clustal Omega, Kalign2, Clustal W2, ProbCons and Probalign respectively. Also, it provides an average TC value of 57.56 *%* representing an improvement of 2.62 *%*, 3.06 *%*, 10.15 *%*, 7.20 *%*, 13.87 *%*, 18.19 *%*, 2.74 *%* and 3.92 *%*, respectively, over that of the other alignment algorithms.

**Table 1:** Average SP and TC values of MSAIndelFR and other multiple alignment algorithms for the benchmarks, BAliBASE 3.0, OXBENCH, PREFAB 4.0 and SABRE (SABmark 1.65)

	BAliBASE		OXBENCH		PREFAB		SABRE	
MSA algorithm	SP (%)	TC (%)	SP (%)	TC (%)	SP (%)	TC (%)	SP (%)	TC (%)
MSAIndelFR	**86.23**	**57.56**	**91.88**	**83.83**	**59.35**	**59.35**	**53.59**	**34.38**
MSAProbs	80.21	(54.93)	(89.39)	(79.78)	(57.52)	(57.52)	(51.55)	(25.21)
MAFFT	84.86	54.50	88.22	77.98	53.93	53.93	50.14	24.33
MUSCLE	82.11	47.41	88.66	78.93	55.74	55.74	46.33	20.80
Clustal Omega	81.94	50.35	88.05	77.76	55.96	55.96	45.11	19.58
Kalign2	80.06	43.68	87.55	77.30	56.33	56.33	41.64	18.91
Clustal W2	75.86	39.37	87.94	77.00	56.05	56.05	40.38	15.98
ProbCons	(85.85)	54.81	88.86	78.80	56.44	56.44	51.27	24.97
Probalign	85.45	53.63	89.08	79.52	56.63	56.63	50.33	23.67

Bold faced values indicate the best performance, while the values in parentheses indicate the second best performance

Boxplots would show more detailed information about the distribution of the SP and TC values than that provided by Table 1. They indicate whether a distribution is skewed or if there are potential unusual observations (outliers) in the data set. In addition, they are very useful when large numbers of test cases are involved and when two or more methods are being compared. Finally, they can be used to determine the first, second (median), and third quartiles as well as interquartile range (IQR) values for various distributions. The width of a box indicates the IQR value, which is the difference between the third and first quartile values.

**Fig. 4:**
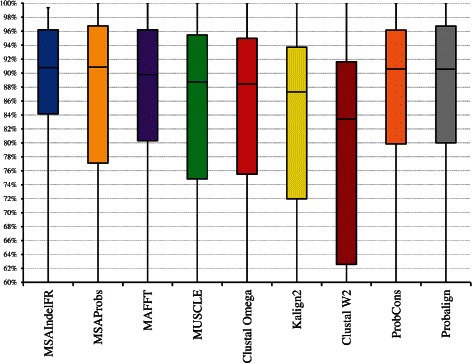
Boxplots for the distributions of the SP values of MSAIndelFR and the other MSA algorithms using the BAliBASE 3.0 benchmark, where the top and bottom of a box and the line in between represent the third quartile, first quartile and median, respectively

**Fig. 5:**
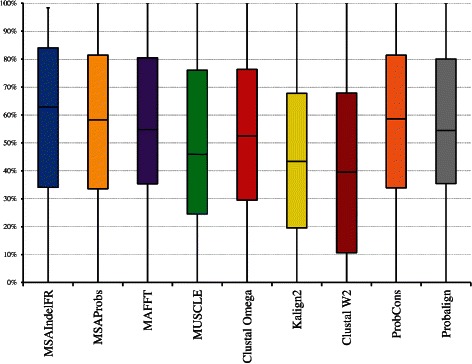
Boxplots for the distributions of the TC values of MSAIndelFR and the other MSA algorithms using the BAliBASE 3.0 benchmark, where the top and bottom of a box and the line in between represent the third quartile, first quartile and median, respectively

In view of the above reasons, boxplots resulting from the distributions of the SP values of the various algorithms evaluated using BAliBASE 3.0 are shown in Fig. 4. This figure clearly shows that MSAIndelFR performs better than the other algorithms, since it has the lowest IQR value as well as the highest first quartile value. It is noted that even though MSAIndelFR, and MSAprobs have an almost equal median value of 91 *%*, the distribution of the SP values generated by MSAIndelFR is much narrower than that generated by MSAProbs, since the former has an IQR value of 12 *%*, whereas the latter a value of 20 *%*. In addition, it is seen that 75 *%* of the MSAIndelFR alignments have an SP value of more than 84 *%* (first quartile), whereas 25 *%* of the alignments have an SP value of more than 96 *%* (third quartile). Figure 5 shows the distributions of the TC values of MSAIndelFR and those of the other algorithms. It is seen from this figure that, just as the case with respect to the SP values, MSAIndelFR performs better than the other algorithms, just as the case is with respect to the SP values.

### Evaluation using OXBENCH

The OXBENCH benchmark [37] is a set of structure-based alignments. Out of the 395 reference alignments in OXBENCH, there are 191 alignments that have protein sequences which belong to one or the other of the 43 selected protein folds.

The average SP and TC values of MSAIndelFR as well as those of the other algorithms using this benchmark as reference are given in Table 1. The results show that MSAIndelFR achieves the highest SP and TC values. Specifically, it provides an average SP value of 91.88 *%* representing an improvement of 2.49 *%*, 3.65 *%*, 3.22 *%*, 3.83 *%*, 4.33 *%*, 3.94 *%*, 3.02 *%* and 2.80 *%* over that of MSAProbs, MAFFT, MUSCLE, Clustal Omega, Kalign2, Clustal W2, ProbCons and Probalign, respectively. Also, it provides an average TC value of 83.83 *%* representing an improvement of 4.05 *%*, 5.85 *%*, 4.90 *%*, 6.07 *%*, 6.53 *%*, 6.83 *%*, 5.02 *%* and 4.31 *%*, respectively, over that of the other alignment algorithms.

The boxplots for the SP and TC value distributions of the various algorithms are given in Additional file 1: Figures S1 and S2, respectively. These figures clearly show that MSAIndelFR performs better than the other algorithms, since it has the lowest IQR value as well as the highest first quartile value. In addition, it is seen that 75 *%* of the MSAIndelFR alignments have an SP value of more than 91 *%* (first quartile), whereas 25 *%* of the alignments have an SP value of 100 *%* (third quartile).

### Evaluation using PREFAB 4.0

The PREFAB 4.0 benchmark [11] is a fully automatically generated benchmark containing 1681 reference alignments. Out of the 1681 reference alignments in PREFAB 4.0, there are 863 alignments that have protein sequences which belong to one or the other of the 43 selected protein folds.

The average SP and TC values of MSAIndelFR as well as those of the other algorithms using this benchmark as reference are given in Table 1. The results show that MSAIndelFR achieves the highest SP and TC values. Specifically, it provides an average SP value of 59.35 *%* representing an improvement of 1.83 *%*, 5.42 *%*, 3.61 *%*, 3.39 *%*, 3.02 *%*, 3.30 *%*, 2.92 *%* and 2.72 *%* over that of MSAProbs, MAFFT, MUSCLE, Clustal Omega, Kalign2, Clustal W2, ProbCons and Probalign, respectively. Also, it provides a similar TC improvements over the other algorithms.

The boxplots for the SP and TC value distributions of the various algorithms are given in Additional file 1: Figures S3 and S4, respectively. These figures clearly show that MSAIndelFR performs better than the other algorithms, since it has the lowest IQR value as well as the highest first quartile value. In addition, it is seen that 75 *%* of the MSAIndelFR alignments have an SP value of more than 31 *%* (first quartile), whereas 25 *%* of the alignments have an SP value of 88 *%* (third quartile).

### Evaluation using SABRE (SABmark 1.65)

The SABmark 1.65 [38] is a very challenging benchmark for multiple sequence alignment. This benchmark is divided into two subsets: Twilight zone and Superfamilies. The similarity level between any two protein sequences is less than 50 *%* in the Superfamily set, while it is at most 25 *%* in the Twilight set. In [41], the author argued that the pairwise reference alignments in SABmark are not suitable to evaluate the MSA algorithms, and hence constructed the SABRE benchmark [42], containing 423 out of the 634 SABmark groups. In this article, we use SABRE instead of the original SABmark benchmark. Out of the 423 reference alignments in the SABRE benchmark, there are 79 alignments that have protein sequences which belong to one or the other of the 43 selected protein folds.

The average SP and TC values of MSAIndelFR as well as those of the other algorithms using this benchmark as reference are given in Table 1. The results show that MSAIndelFR achieves the highest SP and TC values. Specifically, it provides an average SP value of 53.59 *%* representing an improvement of 2.04 *%*, 3.45 *%*, 7.25 *%*, 8.48 *%*, 11.94 *%*, 13.21 *%*, 2.32 *%* and 3.25 *%* over that of MSAProbs, MAFFT, MUSCLE, Clustal Omega, Kalign2, Clustal W2, ProbCons and Probalign, respectively. Also, it provides an average TC value of 34.38 *%* representing an improvement of 9.18 *%*, 10.06 *%*, 13.58 *%*, 14.80 *%*, 15.48 *%*, 18.40 *%*, 9.42 *%* and 10.71 *%*, respectively, over that of the other alignment algorithms.

The boxplots for the SP and TC value distributions of the various algorithms are given in Additional file 1: Figures S5 and S6, respectively. These figures clearly show that even for this challenging benchmark, MSAIndelFR performs better than all the other algorithms in terms of the median value (52 *%*). In addition, it is seen that 75 *%* of MSAIndelFR alignments have an SP value of more than 29 *%* (first quartile), whereas 25 *%* of the alignments have an SP value of more than 77 *%* (third quartile).

### Statistical significance

The Wilcoxon matched-pair signed-rank test [40] is now used to determine if the improvements achieved, in terms of the SP and TC values, by the proposed MSAIndelFR algorithm are statistically significant. Tables 2 and 3 give the *p*-values obtained by the Wilcoxon matched-pair signed-rank test between the proposed MSAIndelFR and other alignment algorithms for the four benchmarks using the SP and TC scores, respectively. A *p*-value less than 0.05 is considered to be statistically significant [8, 12, 13]. Thus, it is seen from Table 2 that MSAIndelFR yields improvements that are statistically very significant over all the other algorithms on the BAliBASE and PREFAB benchmarks, as far as the SP values are concerned. It also achieves statistically significant improvements over five of the algorithms, MAFFT, MUSCLE, Clustal Omega, Kalign2 and Clustal W2 on the OXBENCH and SABRE benchmarks. As to the improvement achieved in term of the TC values, it seen from Table 3 that MSAIndelFR achieves, in general, statistically significant improvements over the algorithms, MAFFT, MUSCLE, Clustal Omega, Kalign2 and Clustal W2 on all the four benchmarks.

**Table 2:** *P*-values obtained by the Wilcoxon matched-pair signed-rank test between MSAIndelFR and the other multiple alignment algorithms on the benchmarks, BAliBASE 3.0, OXBENCH, PREFAB 4.0 and SABRE (SABmark 1.65) using SP scores

MSA algorithm	BAliBASE	OXBENCH	PREFAB	SABRE
MSAProbs	4.52×10^−3^	0.128	3.2×10^−3^	0.344
MAFFT	2.82×10^−8^	1.78×10^−7^	9.23×10^−5^	4.24×10^−2^
MUSCLE	2.57×10^−11^	9.32×10^−4^	7.7×10^−8^	1.0×10^−2^
Clustal Omega	2.51×10^−14^	1.96×10^−5^	5.4×10^−5^	3.65×10^−2^
Kalign2	1.79×10^−7^	1.63×10^−6^	1.06×10^−6^	1.2×10^−4^
Clustal W2	7.76×10^−17^	3.4×10^−5^	3.54×10^−7^	3.25×10^−5^
ProbCons	5.49×10^−3^	0.243	6.17×10^−8^	0.398
Probalign	5.67×10^−3^	0.215	4.70×10^−9^	0.388

**Table 3:** *P*-values obtained by the Wilcoxon matched-pair signed-rank test between MSAIndelFR and the other multiple alignment algorithms on the benchmarks, BAliBASE 3.0, OXBENCH, PREFAB 4.0 and SABRE (SABmark 1.65) using TC scores

MSA algorithm	BAliBASE	OXBENCH	PREFAB	SABRE
MSAProbs	6.09×10^−2^	0.298	3.2×10^−3^	0.125
MAFFT	6.97×10^−7^	1.20×10^−5^	9.23×10^−5^	1.86×10^−2^
MUSCLE	2.18×10^−11^	7.41×10^−3^	7.7×10^−8^	5.10×10^−2^
Clustal Omega	4.11×10^−8^	1.29×10^−4^	5.4×10^−5^	8.47×10^−3^
Kalign2	5.46×10^−6^	1.36×10^−6^	1.06×10^−6^	3.99×10^−3^
Clustal W2	3.86×10^−11^	3.47×10^−6^	3.54×10^−7^	2.50×10^−4^
ProbCons	2.48×10^−2^	0.694	6.17×10^−8^	0.288
Probalign	8.92×10^−2^	0.377	4.70×10^−9^	0.147

### Run time comparison

We now compare the run times of the proposed MSAIndelFR and other alignment algorithms using a desktop PC with Intel(R) Core(TM) i7–2600 CPU at 3.40GHZ and RAM of 16GB. As explained earlier, MSAIndelFR needs the computed average log–loss values and the predicted locations of IndelFRs to set the gap penalty functions for each protein sequence in the selected reference alignments from the four benchmarks (see Eqs. (2) and (3)). This information is available in [43]. The alignment times (in seconds) of the MSAIndelFR and other algorithms for aligning the protein sequences from the four alignment benchmarks are given in Table 4. It is seen from this table that the proposed MSAIndelFR algorithm provides the second best alignment time after Kalign2, but outperforms Kalign2 in terms of both the SP and TC metrics for all the benchmarks.

**Table 4:** Overall execution time (in seconds) of MSAIndelFR and other multiple alignment algorithms using the benchmarks, BAliBASE 3.0, OXBENCH, PREFAB 4.0 and SABmark 1.65

MSA algorithm	BAliBASE	OXBENCH	PREFAB	SABRE
MSAIndelFR	(131.63)	(9.38)	(35.59)	(6.51)
MSAProbs	1323.47	14.9	44.49	17.52
MAFFT	1270.66	333.58	1511.83	155.37
MUSCLE	665.11	94.35	28.92	60.58
Clustal Omega	199.86	12.07	40.7	10.13
Kalign2	**32.74**	**7.54**	**32.9**	**3.66**
Clustal W2	769.35	12.55	35.69	10.69
ProbCons	7526	45.22	76.90	65.10
Probalign	4623	25.40	58.38	30.59

Bold faced values indicate the best performance, while the values in parentheses indicate the second best performance

## Conclusion

In this article, we have proposed a novel and efficient algorithm, MSAIndelFR algorithm, for multiple protein sequence alignment; the algorithm incorporates the information on the predicted locations of IndelFRs and the computed average log–loss values obtained from IndelFR predictors, each of which is designed for a different protein fold. A new variable gap penalty function has been proposed to make the gap placement more accurate in the protein alignment, wherein the gap opening penalty is position–specific and the gap extension penalty is region–specific. In order to study the performance of the proposed algorithm, an extensive evaluation has been carried using some of the protein sequences from the four popular benchmarks, namely, BAliBASE 3.0, OXBENCH, PREFAB 4.5, and SABRE (SABmark 1.65). In this selection of these sequences, it is ensured that they belong to one of the 43 protein folds for which IndelFR predictors are available. The results have shown that the performance of the proposed MSAIndelFR algorithm is superior to that of the eight most–widely used alignment algorithms, Clustal W2, Clustal Omega, MSAProbs, Kalign2, MAFFT, MUSCLE, ProbCons and Probalign, in terms of both the SP and TC metrics which have been calculated using reference alignments of the four benchmarks. Furthermore, it has been shown that the improvements achieved over all the other algorithms by the proposed algorithm are, in general, statistically significant. It is to be made clear that the concepts behind the proposed alignment algorithm are not restricted to the 43 protein folds considered in this article. These protein folds have been used to illustrate the proposed algorithm. However, if a protein sequence to be aligned belongs to some other protein fold, a new predictor needs to be first constructed and then used in the proposed alignment scheme.

## Availability of supporting data

The source code is available on request from the authors.

## Endnote

^1^ An example of calculating the SP and TC values is given in Section 2 of the Additional file 1.

## Additional file

Additional file 1 **Supplementary materials.** Additional file 1 contains more details about the modified version of FASTA model, example explains how both the sum–of–pairs (SP) and the total column (TC) values are computed, Boxplots of SP and TC value distributions of the MSAIndelFR and other MSA algorithms using OXBENCH, PREFAB and SABRE (SABmark) benchmarks, and the list of the 43 protein folds from the three different protein classes. (PDF 2703 kb)

## Abbreviations

AGP: Affine gap penalty
DP: Dynamic programming
GPE: Gap extension penalty
GPO: Gap opening penalty
indel: Insertion or deletion mutation
IndelFR: Indel flanking region
IndelFRs: Indel flanking regions
LPPM: Left PPM
MSA: Multiple sequence alignment
OF: Objective function
PPM: Prediction by partial match
RPPM: Right PPM
SP: Sum–
of–pairs; TC: Total columns
UPGMA: Unweighted pair group method with arithmetic mean
VGP: Variable gap penalty
VOMM: Variable–order Markov model
