# Supplementary material for: MSAIndelFR: a scheme for multiple protein sequence alignment using information on indel flanking regions
Source: BMC Bioinformatics. 2015 Nov 23;16:393. doi: 10.1186/s12859-015-0826-3 (PMC4657235; doi:10.1186/s12859-015-0826-3)
Supplement: Supplementary file 1 — Supplementary materials. Additional file 1 contains more details about the modified version of FASTA model, example explains how both the sum–of–pairs (SP) and the total column (TC) values are computed, Boxplots of SP and TC value distributions of the MSAIndelFR and other MSA algorithms using OXBENCH, PREFAB and SABRE (SABmark) benchmarks, and the list of the 43 protein folds from the three different protein classes. (PDF 2703 kb) [file 12859_2015_826_MOESM1_ESM.pdf]

Supplementary Material

1 Modified version of FASTA

The modified version of FASTA format is the same as the standard FASTA format except that the *position-specific gap opening penalties* and the predicted IndelFRs are included after the main list of amino acids of a given protein sequence. The position-specific gap opening penalties are enclosed by curly brackets { } and indicated by the label "GPO:". The predicted IndelFRs are included in three separate lines and enclosed by curly brackets { } : one for the start location of the left flanking regions, one for the start location of the right flanking regions, and one for the complete IndelFRs. The start locations of the left and right flanking regions are indicated by "LLOC:" and "RLOC:", respectively, while the complete IndelFRs by "IndelFRs". An example is given below for the protein sequences, "*1hjd_A*", "*1pht_*" and "*1h1c_A*" of length 101, 83 and 329, respectively, written using the modified version of FASTA.

>1hjd_A

ADRKLCADQECSHPISMAVALQDYMAPDCRFLTIHRGQVVYVFSKLKGRGRLFWGGSVQGDYYG

DLAARLGYFPSSIVREDQTLKPGKVDVKTDKWDFYCQ

{GPO: 8.555 8.788 8.345 7.982 7.448 8.230 8.003 8.146 7.328
7.101 7.772 7.211 7.486 6.681 5.500 4.650 3.804 3.095 2.076
3.393 3.481 4.052 4.703 5.419 4.775 3.749 2.838 1.802 2.633
3.924 4.803 5.259 5.987 5.221 4.348 3.465 2.354 1.475 3.059
4.008 4.953 4.942 5.388 7.387 6.451 5.501 4.468 4.721 4.445
3.684 3.545 4.444 5.085 4.599 4.323 3.972 3.883 5.886 6.325
5.669 4.973 5.349 5.418 4.978 4.826 4.952 5.250 5.545 6.498
6.273 6.104 6.729 7.094 7.917 7.465 7.379 7.841 6.890 6.547
6.711 7.057 7.263 7.778 7.959 8.226 8.271 8.558 8.752 8.449
8.714 8.476 8.757 8.757 8.757 8.757 8.757 8.757 8.757 8.757
8.757 8.757}

{LLOC: 19 38 57}

{RLOC: 28 51 65}

{IndelFR: 19 – 76}

>1pht_

AEGYQYRALYDYKKEREEDIDLHLGDILTVNKGSLVALGFSDGQEARPEEIGWLNGYNETTGER

GDFPGTYVEYIGRKKISPP

{GPO: 6.694 6.724 6.247 6.688 6.634 6.607 6.326 6.708 6.859 7.013 7.262 7.528 7.105 7.755 8.015 7.748 7.462 7.023 6.331 5.660 6.188 5.987 6.116 5.651 5.764 6.484 6.775 6.017 6.314 6.433 6.293 7.209 7.436 7.247 7.418 6.644 7.878 7.583 8.145 7.605 6.913 6.096 6.604 6.463 6.025 6.181 6.071 6.290 5.767 6.715 7.267 7.266 7.030 6.751 6.783 7.131 6.992 6.941 6.842 6.813 7.020 7.320 7.783 7.675 7.426 7.058 6.652 6.652 6.796 6.707 7.131 7.176 7.605 7.346 7.346 7.346 7.346 7.346 7.346 7.346 7.346 7.346 7.346 }

{LLOC: 24 60}

{RLOC: 31 67}

{IndelFRs: 24-41, 60-77}

>1h1c_A

LIAKRAYPYETEKRDKTYLALNENPFPFPEDLVDEVFRRLNSDALRIYYDSPDEELIEKILSYL

DTDFLSKNNVSVGNGADEIIYVXXLXFDRSVFFPPTYSCYRIFAKAVGAKFLEVPLTKDLRIPE

VNVGEGDVVFIPNPNNPTGHVFEREEIERILKTGAFVALDEAYYEFHGESYVDFLKKYENLAVI

RTFSKAFSLAAQRVGYVVASEKFIDAYNRVRLPFNVSYVSQXFAKVALDHREIFEERTKFIVEE

RERXKSALREXGYRITDSRGNFVFVFXEKEEKERLLEHLRTKNVAVRSFREGVRITIGKREEND

XILRELEVF

{GPO: 7.819 7.668 8.075 7.884 7.457 6.907 6.558 6.168 6.691

6.018 5.032 4.630 5.180 6.245 6.763 6.873 7.007 7.243 7.626

7.794 7.517 7.643 7.455 7.416 7.461 7.398 6.719 7.068 7.067

6.932 6.690 6.989 6.567 7.098 6.870 6.737 6.719 6.648 7.186

7.390 7.383 7.238 6.885 7.010 6.534 6.532 6.022 6.267 6.011

6.524 6.882 6.129 6.587 6.892 6.235 6.810 6.356 6.604 6.930

6.181 6.626 7.007 7.029 6.844 6.166 6.482 7.217 6.738 7.316

7.476 7.114 6.792 6.867 6.680 6.399 6.384 5.752 7.353 6.822

6.182 6.902 6.593 6.719 7.043 7.240 6.558 6.280 7.418 7.130

6.688 7.643 6.906 7.381 7.211 8.012 7.816 7.133 7.596 7.479

7.842 7.087 6.947 7.184 6.672 6.736 7.274 6.927 6.439 6.878

7.422 6.960 7.097 7.320 7.350 7.197 7.474 7.256 7.402 7.095

6.871 7.112 6.805 6.754 6.166 5.509 5.111 5.551 4.945 5.238

5.828 5.996 7.231 7.386 8.140 7.849 7.893 8.102 8.260 8.309

8.665 8.462 8.440 7.877 7.669 7.090 7.387 7.295 7.377 6.848

6.393	6.303	6.793	6.569	5.847	5.968	6.519	6.126	6.113	6.085
6.724	7.338	7.656	7.734	7.904	7.882	7.914	7.169	7.663	7.686
7.207	7.352	7.339	6.812	6.738	6.412	5.471	5.643	5.941	6.040
5.627	5.311	6.813	6.404	6.770	6.982	7.173	6.481	6.957	6.701
6.641	7.257	6.888	7.238	6.669	7.139	6.857	6.106	6.380	6.472
5.993	6.355	6.033	6.075	7.467	7.352	7.101	7.097	6.813	6.877
7.634	7.561	7.073	6.788	7.461	6.805	6.748	7.525	7.492	6.783
6.425	6.571	6.024	6.387	6.168	7.599	7.155	7.405	7.027	7.526
7.415	6.957	7.081	7.722	7.398	7.496	7.103	6.985	6.058	6.722
6.324	6.654	6.519	6.678	6.384	6.727	6.951	6.710	6.950	7.098
7.129	7.898	7.518	7.092	7.161	6.509	6.908	6.460	8.010	7.712
6.895	7.235	6.434	6.343	7.164	6.978	7.694	7.119	7.720	7.493
7.996	7.835	7.541	7.796	8.621	8.140	7.698	7.228	7.482	6.732
7.361	6.868	7.025	6.566	7.147	7.375	7.250	7.447	7.552	7.373
7.572	7.513	7.340	7.151	7.290	7.541	7.493	7.032	7.563	7.259
6.221	6.291	5.632	5.755	6.272	6.267	6.101	6.659	6.341	6.968
6.599	5.697	7.371	6.746	6.399	5.617	5.876	5.477	5.597	7.128

7.268 7.268 7.268 7.268 7.268 7.268 7.268 7.268 7.268 7.268 }

{LLOC: 33 77 128 194 222 255}

{RLOC: 38 90 145 200 231 263}

{IndelFRs: 33-48, 77-100, 128-155, 194-210, 222-241, 255-273}

2 Accuracy of Alignment

Example: In this example, we explain how the *sum-of-pairs* (SP) and the *total column* (TC) values are computed.

Given the reference Alignment:

```
Seq [0]: GKGDRKK
Seq [1]: MQ-DRVK
Seq [2]: MKKLKKH
Seq [3]: MHIK-PL
```

and the test Alignment:

```
Seq [0]: GK-GDRKK
Seq [1]: MQ-DRVK-
Seq [2]: MKKLKKH-
Seq [3]: MHIK-PL-
```

the *sum-of-pairs* (SP) value is given by

$$SP = \frac{\textit{The number of correctly aligned amino acids pairs found in the test alignment}}{\textit{The total number of aligned amino acids pairs in the reference alignment}}$$

and the *total column* (TC) value is given by

$$TC = \frac{\textit{The number of correctly aligned columns found in the test alignment}}{\textit{The total number of aligned columns in the reference alignment}}$$

Step-by-step calculation of the intermediate values required for the computation of SP and TC

Reference Alignment Column	Aligned amino acids pair count in reference alignment	Correctly aligned column count in test alignment	Correctly aligned amino acids pair count in test alignment
1	$\frac{4(4-1)}{2}=6$	1	$\frac{4(4-1)}{2}=6$
2	$6+\frac{4(4-1)}{2}=12$	2	$6+\frac{4(4-1)}{2}=12$
3	$12+\frac{3(3-1)}{2}=15$	2	$12+\frac{2(2-1)}{2}=13$
4	$15+\frac{4(4-1)}{2}=21$	2	$13+\frac{3(3-1)}{2}=16$
5	$21+\frac{3(3-1)}{2}=24$	2	$16+\frac{2(2-1)}{2}=17$
6	$24+\frac{4(4-1)}{2}=30$	2	$17+\frac{3(3-1)}{2}=20$
7	$30+\frac{4(4-1)}{2}=36$	2	$20+\frac{3(3-1)}{2}=23$

SP and TC values can be computed by substituting the values given in the last row of the above table.

$$\boldsymbol{SP} = \frac{23}{36} = 0.639$$

$$\boldsymbol{TC} = \frac{2}{7} = 0.286$$

3 Supplementary figures

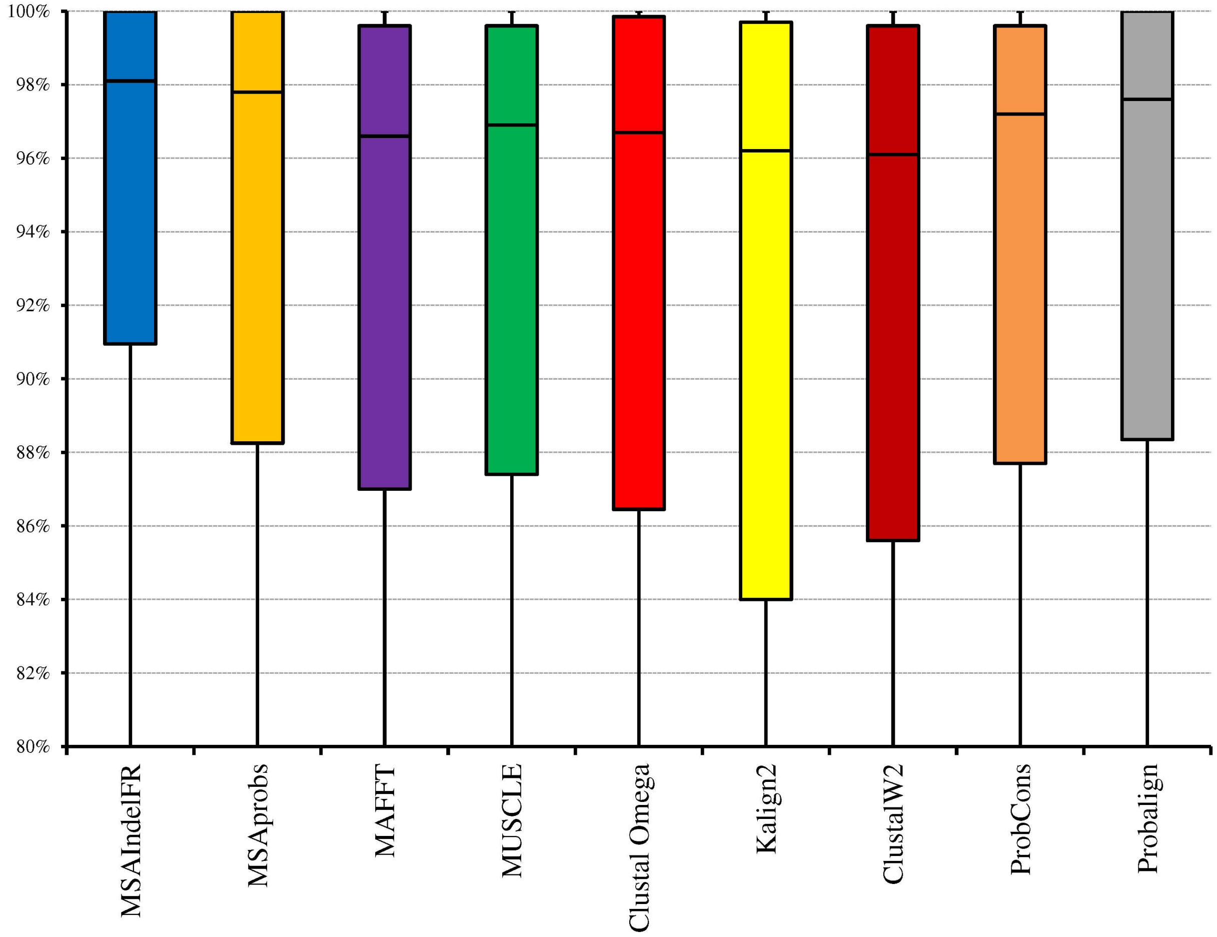

Figure S 1. Boxplots for the distributions of the SP values of MSAIndelFR and the other MSA algorithms using the OXBENCH benchmark, where the top and bottom of a box and the line in between represent the third quartile, first quartile and median, respectively.

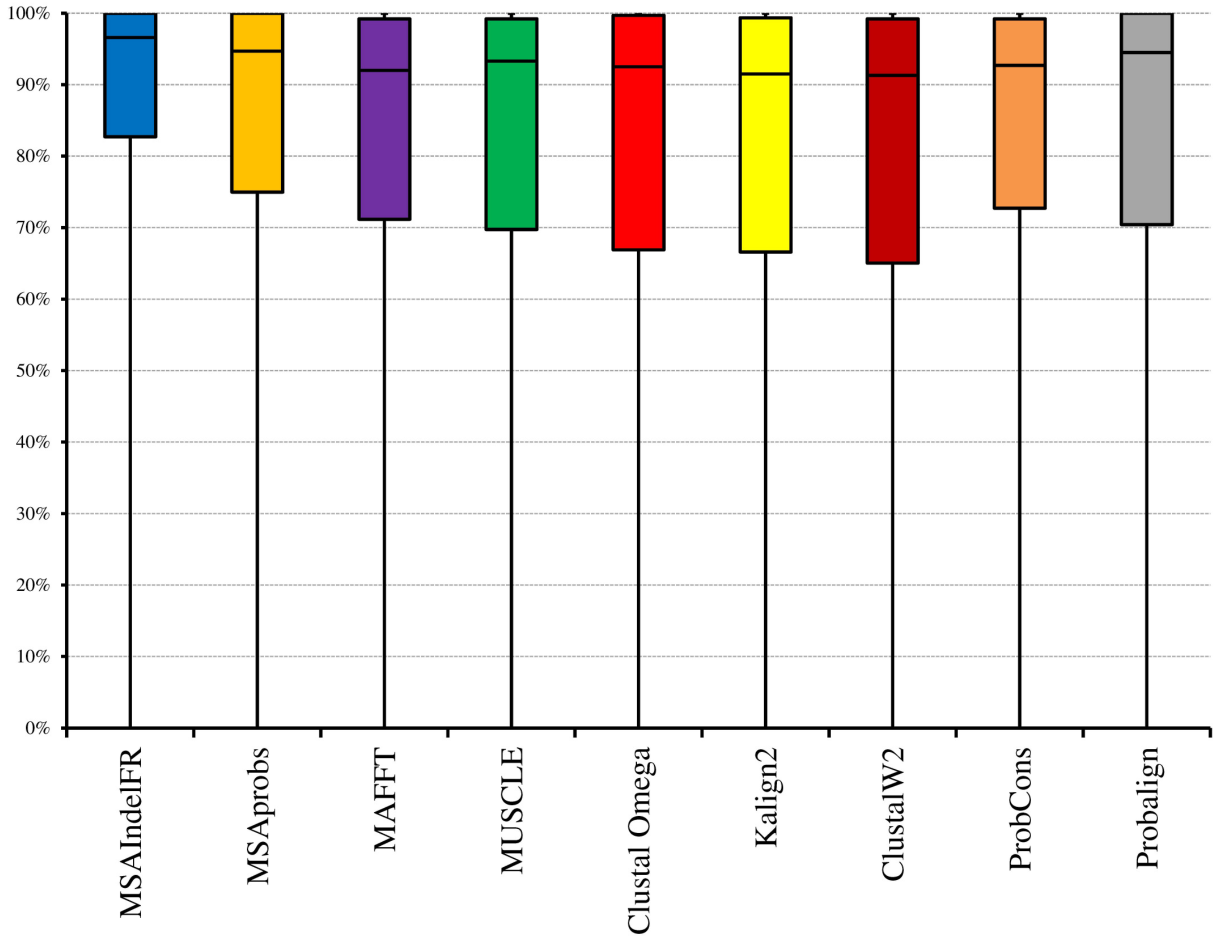

Figure S 2. Boxplots for the distributions of the TC values of MSAIndelFR and the other MSA algorithms using the OXBENCH benchmark, where the top and bottom of a box and the line in between represent the third quartile, first quartile and median, respectively.

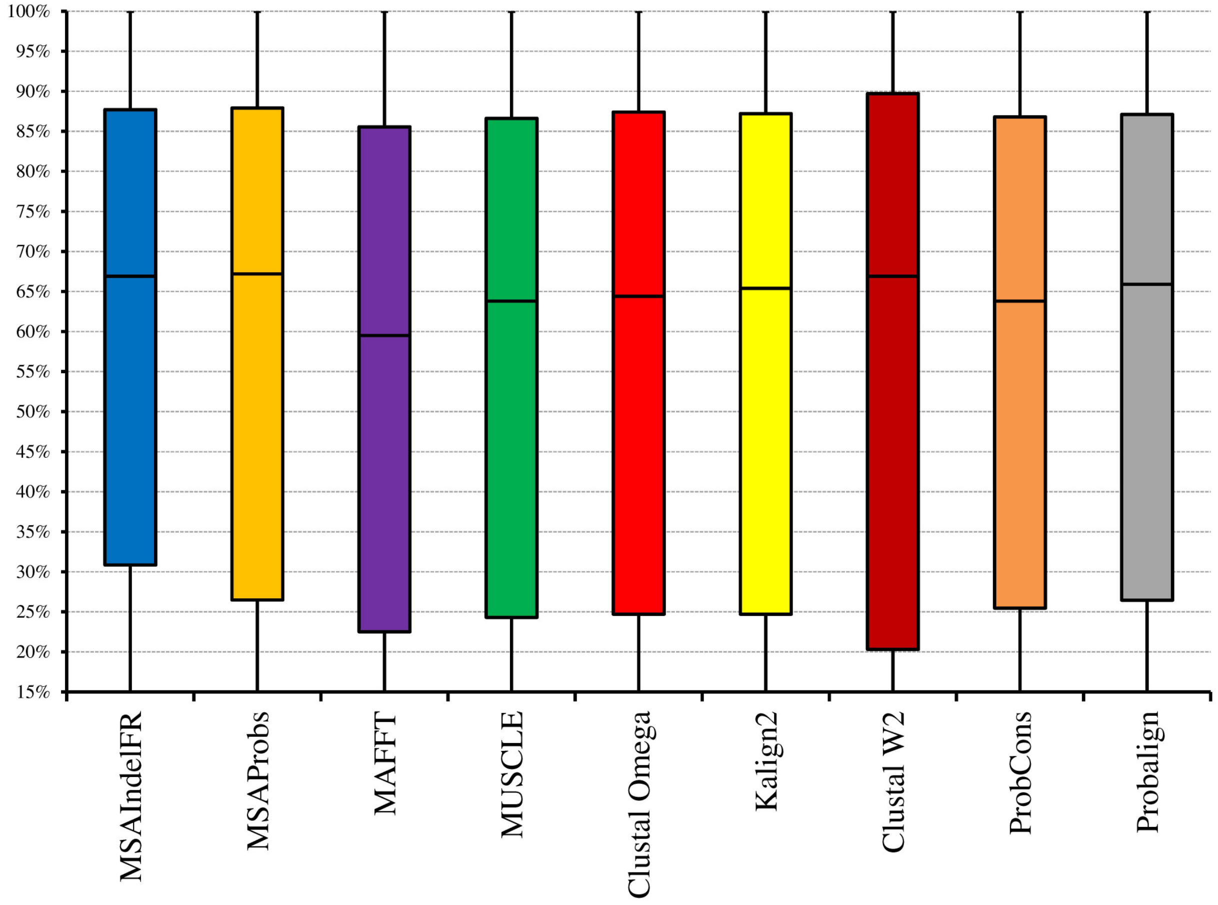

Figure S 3. Boxplots for the distributions of the SP values of MSAIndelFR and the other MSA algorithms using the PREFAB benchmark, where the top and bottom of a box and the line in between represent the third quartile, first quartile and median, respectively.

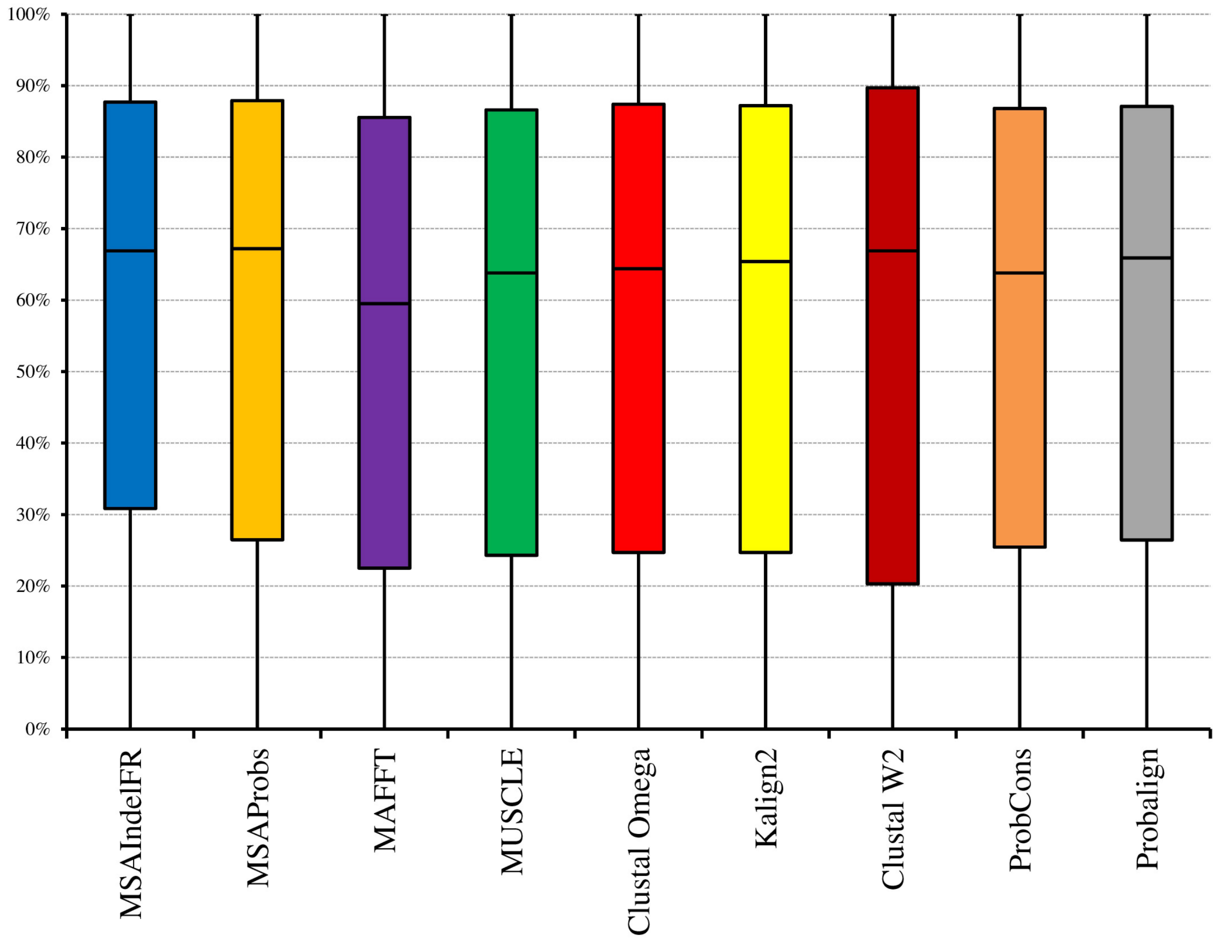

Figure S 4. Boxplots for the distributions of the TC values of MSAIndelFR and the other MSA algorithms using the PREFAB benchmark, where the top and bottom of a box and the line in between represent the third quartile, first quartile and median, respectively.

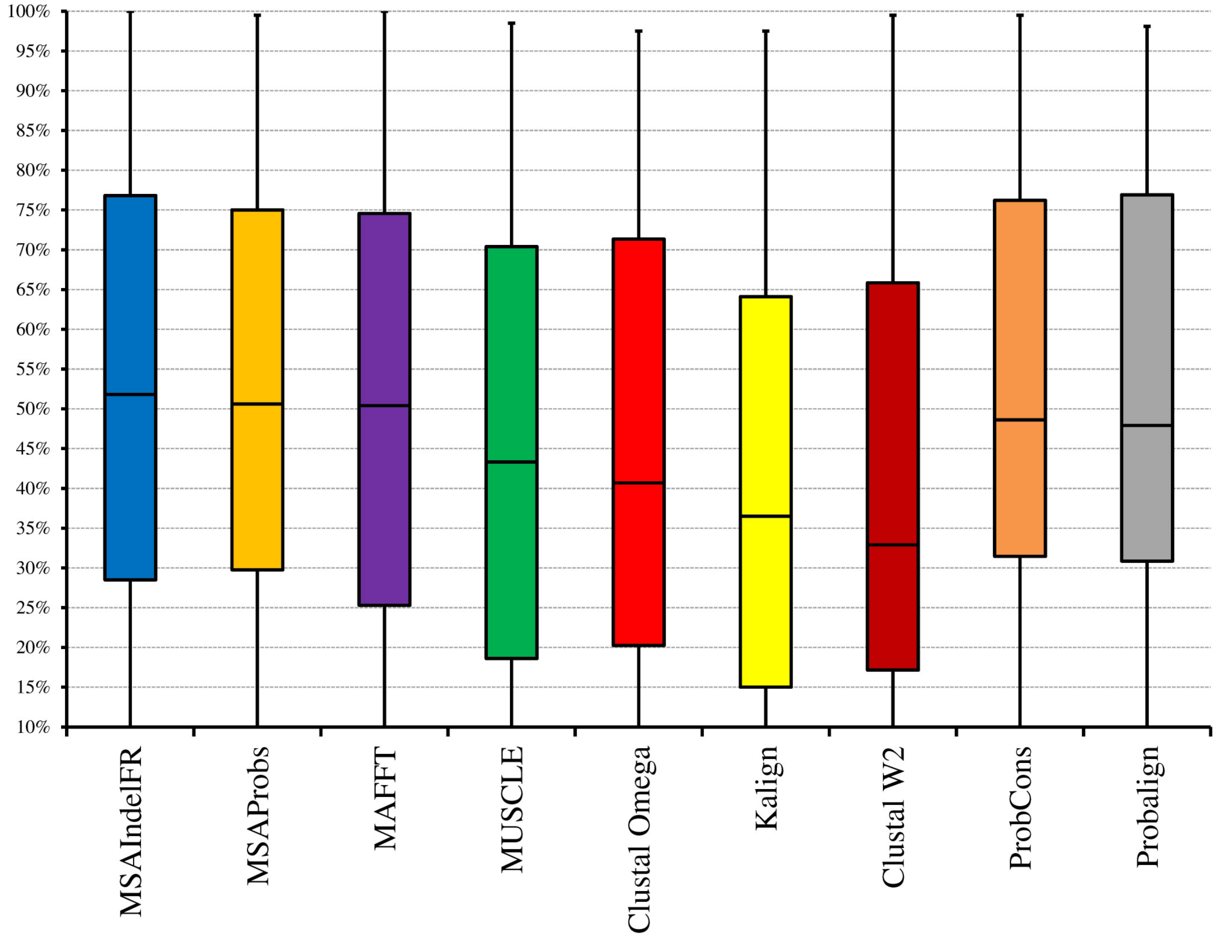

Figure S 5. Boxplots for the distributions of the SP values of MSAIndelFR and the other MSA algorithms using the (SABRE) SABmark benchmark, where the top and bottom of a box and the line in between represent the third quartile, first quartile and median, respectively.

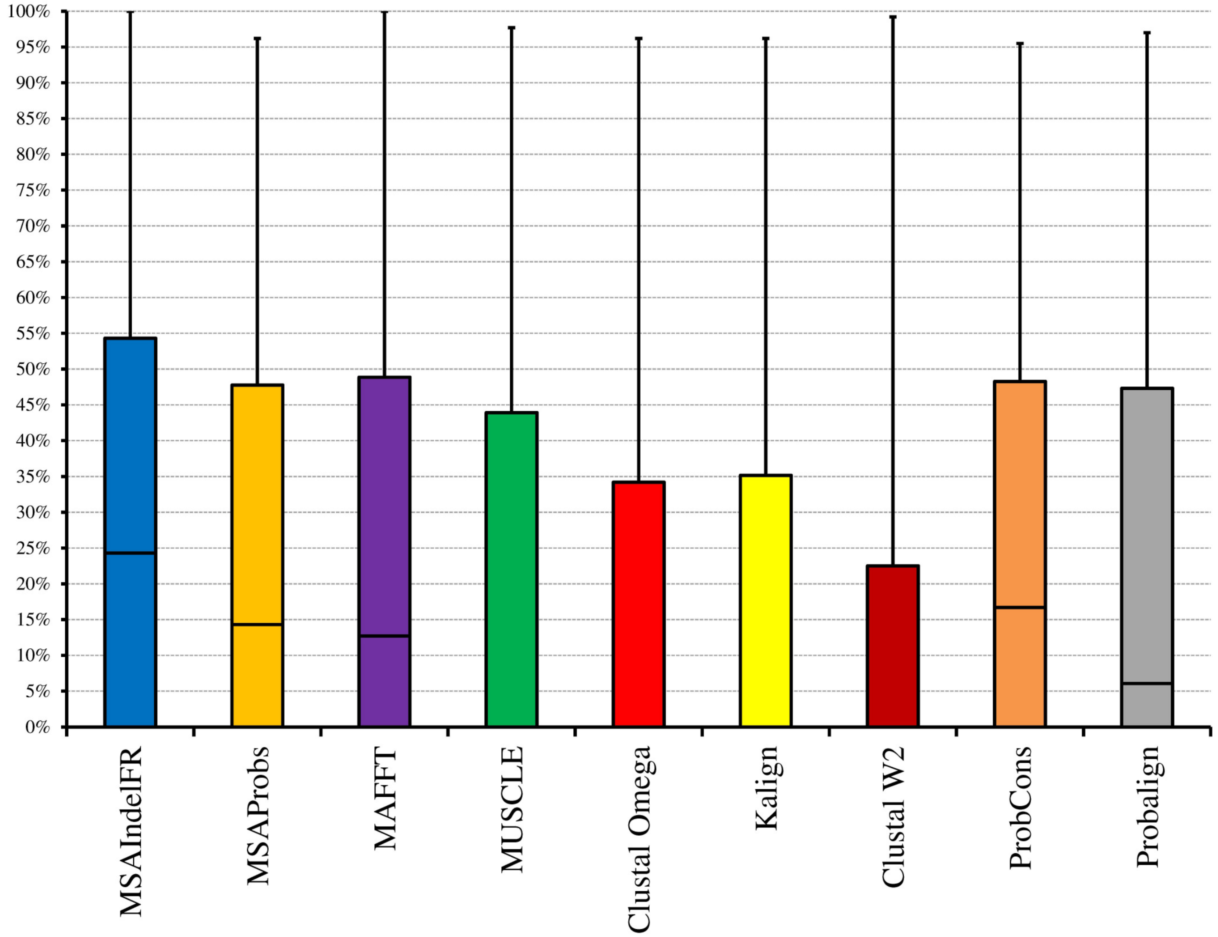

Figure S 6. Boxplots for the distributions of the TC values of MSAIndelFR and the other MSA algorithms using the (SABRE) SABmark benchmark, where the top and bottom of a box and the line in between represent the third quartile, first quartile and median, respectively.

4 Supplementary tables

Table S 1: Protein folds from the *All-* α *protein* class

label	Protein folds	Protein superfamilies
**A1**	a.1: Globin-like	a.1.1: Globin-like
**A3**	a.3: Cytochrome c	a.3.1: Cytochrome c
**A4**	a.4: DNA/RNA-binding 3-helical bundle	a.4.1: Homeodomain-like<br>a.4.5: "Winged helix" DNA-binding domain
**A22**	a.22: Histone-fold	a.22.1: Histone-fold
**A25**	a.25: Ferritin-like	a.25.1: Ferritin-like
**A26**	a.26: 4-helical cytokines	a.26.1: 4-helical cytokines
**A35**	a.35: lambda repressor-like DNA-binding domains	a.35.1: lambda repressor-like DNA-binding domains
**A39**	a.39: EF Hand-like	a.39.1: EF-hand
**A45**	a.45: Glutathione S-transferase (GST), C-terminal domain	a.45.1: Glutathione S-transferase (GST), C-terminal domain
**A118**	a.118: alpha-alpha superhelix	a.118.1: ARM repeat<br>a.118.3: Sec7 domain<br>a.118.7: 14-3-3 protein<br>a.118.9: ENTH/VHS domain<br>a.118.8: TPR-like
**A133**	a.133: Phospholipase A2, PLA2	a.133.1: Phospholipase A2, PLA2

Table S 2: Protein folds from the *All-* β *protein* class

label	Protein folds	Protein superfamilies
**B6**	b.6: Cupredoxin-like	b.6.1: Cupredoxins
**B18**	b.18: Galactose-binding domain-like	b.18.1: Galactose-binding domain-like
**B29**	b.29: Concanavalin A-like lectins/glucanases	b.29.1: Concanavalin A-like lectins/glucanases
**B34**	b.34: SH3-like barrel	b.34.2: SH3-domain
**B35**	Fold b.35: GroES-like	b.35.1: GroES-like
**B36**	b.36: PDZ domain-like	b.36.1: PDZ domain-like
**B40**	b.40: OB-fold	b.40.5: Inorganic pyrophosphatase<br>b.40.1: Staphylococcal nuclease<br>b.40.6: MOP-like
**B42**	b.42: beta-Trefoil	b.42.1: Cytokine<br>b.42.2: Ricin B-like lectins
**B47**	b.47: Trypsin-like serine proteases	b.47.1: Trypsin-like serine proteases
**B50**	b.50: Acid proteases	b.50.1: Acid proteases
**B55**	b.55: PH domain-like	b.55.1: PH domain-like
**B60**	b.60: Lipocalins	b.60.1: Lipocalins
**B82**	b.82: Double-stranded beta-helix	b.82.1: RmlC-like cupins<br>b.82.3: cAMP-binding domain-like
**B121**	b.121: Nucleoplasmin-like/VP (viral coat and capsid proteins)	b.121.2: Group II dsDNA viruses VP<br>b.121.4: Positive stranded ssRNA viruses

Table S 3: Protein folds from the α *and* β *proteins (a/b)* class

label	Protein folds	Protein superfamilies
**C1**	c.1: TIM beta/alpha-barrel	c.1.1: Triosephosphate isomerase (TIM)<br>c.1.2: Ribulose-phoshate binding barrel<br>c.1.3: Thiamin phosphate synthase<br>c.1.4: FMN-linked oxidoreductases<br>c.1.5: Inosine monophosphate dehydrogenase (IMPDH)<br>c.1.6: PLP-binding barrel<br>c.1.7: NAD(P)-linked oxidoreductase<br>c.1.8: (Trans)glycosidases<br>c.1.9: Metallo-dependent hydrolases<br>c.1.10: Aldolase<br>c.1.11: Enolase C-terminal domain-like<br>c.1.12: Phosphoenolpyruvate/pyruvate domain<br>c.1.13: Malate synthase G<br>c.1.14: RuBisCo, C-terminal domain<br>c.1.15: Xylose isomerase-like<br>c.1.16: Bacterial luciferase-like<br>c.1.17: Nicotinate/Quinolinate PRTase C-terminal domain-like<br>c.1.18: PLC-like phosphodiesterases<br>c.1.19: Cobalamin (vitamin B12)-dependent enzymes<br>c.1.20: tRNA-guanine transglycosylase<br>c.1.21: Dihydropteroate synthetase-like<br>c.1.26: Homocysteine S-methyltransferase<br>c.1.27: (2r)-phospho-3-sulfolactate synthase ComA
**C2**	c.2: NAD(P)-binding Rossmann-fold domains	c.2.1: NAD(P)-binding Rossmann-fold domains
**C3**	c.3: FAD/NAD(P)-binding domain	c.3.1: FAD/NAD(P)-binding domain
**C14**	c.14: ClpP/crotonase	c.14.1: ClpP/crotonase
**C23**	c.23: Flavodoxin-like	c.23.1: CheY-like<br>c.23.5: Flavoproteins<br>c.23.6: Cobalamin (vitamin B12)-binding domain<br>c.23.8: N5-CAIR mutase (phosphoribosylaminoimidazole carboxylase, PurE)<br>c.23.12: Formate/glycerate dehydrogenase catalytic domain-like<br>c.23.13: Type II 3-dehydroquinate dehydratase<br>c.23.14: N-(deoxy)ribosyltransferase-like<br>c.23.16: Class I glutamine amidotransferase-like
**C26**	c.26: Adenine nucleotide alpha hydrolase-like	c.26.1: Nucleotidylyl transferase<br>c.26.2: Adenine nucleotide alpha hydrolases-like
**C36**	c.36: Thiamin diphosphate-binding fold (THDP-binding)	c.36.1: Thiamin diphosphate-binding fold (THDP-binding)
**C37**	c.37: P-loop containing nucleoside triphosphate hydrolases	c.37.1: P-loop containing nucleoside triphosphate hydrolases
**C47**	c.47: Thioredoxin fold	c.47.1: Thioredoxin-like
**C55**	c.55: Ribonuclease H-like motif	c.55.1: Actin-like ATPase domain<br>c.55.2: Creatinase/prolidase N-terminal domain<br>c.55.3: Ribonuclease H-like
**C56**	c.56: Phosphorylase/hydrolase-like	c.56.2: Purine and uridine phosphorylases<br>c.56.5: Zn-dependent exopeptidases
**C61**	c.61: PRTase-like	c.61.1: PRTase-like
**C67**	c.67: PLP-dependent transferase-like	c.67.1: PLP-dependent transferases
**C68**	c.68: Nucleotide-diphospho-sugar transferases	c.68.1: Nucleotide-diphospho-sugar transferases
**C69**	c.69: alpha/beta-Hydrolases	c.69.1: alpha/beta-Hydrolases
**C94**	c.94: Periplasmic binding protein-like II	c.94.1: Periplasmic binding protein-like II
**C95**	c.95: Thiolase-like	c.95.1: Thiolase-like
**C108**	c.108: HAD-like	c.108.1: HAD-like

5 The training sets of IndelFR predictor and test set of MSAIndelFR algorithm

In the BAliBASE 3.0 benchmark, we have selected 186 reference alignments that use 2064 different protein sequences. The distribution of these protein sequences over the protein folds is given in Table A1. We have determined that only 13 of these 2064 protein sequences were involved in training the PPM IndelFR predictors. The distribution of these 13 protein sequences over the protein folds is listed in Table A2.

Table A1

Protein folds	**A.3**	**A.4**	**A.35**	**A.45**	**B.6**	**B.18**	**B.29**	**B.34**
Number of sequence	78	113	8	15	36	34	4	64
Protein folds	**B.35**	**B.40**	**B.47**	**B.50**	**B.121**	**C.1**	**C.2**	**C.3**
Number of sequence	2	11	148	10	17	361	268	140
Protein folds	**C.23**	**C.26**	**C.37**	**C.47**	**C.61**	**C.67**	**C.69**	**C.94**
Number of sequence	9	26	213	201	30	187	60	29

Note: A1, A3, A4, A22, A25, A26, A35, A39, A45, A118, A133, B6, B18, B29, B34, B35, B36, B40, B42, B47, B50, B55, B60, B82, B121, C1, C2, C3, C14, C23, C26, C36, C37, C47, C55, C56, C61, C67, C68, C69, C94, C95 and C108 are the protein folds (See Additional file 1- Tables S1–S3)

Table A2

Protein folds	A.3	B.6	B.34	B.121	C.2	C.26	C.37	C.47	C.94
Number of sequence	1	1	1	1	4	1	1	2	1

It can be seen from the above statistics that for 15 of the PPM IndelFR predictors out of the 24 predictors, there is no protein sequence that is common between their training sets and the test set for the proposed MSAIndelFR algorithm. It should be pointed out that for any of the predictors for the remaining 9 protein folds, the maximum overlap between the training set and the test set is less than 5.9%.

In the OXBENCH benchmark, we have selected 191 reference alignments that use 405 different protein sequences. The distribution of these protein sequences over the protein folds is given in Table B1. We have determined that only 10 of these 405 protein sequences were involved in training the PPM IndelFR predictors. The distribution of these 10 protein sequences over the protein folds is listed in Table B2.

Table B1

Protein folds	A.1	A.3	A.4	A.25	A.26	A35	A.39	A.45	B.6	B.18	B.29
Number of sequence	35	18	7	5	5	4	21	1	11	1	16

Protein folds	B.34	B.35	B.40	B.42	B.47	B.50	B.60	C.1	C.2	C.3	C.23
Number of sequence	10	4	11	13	5	33	14	53	44	17	10

Protein folds	C.37	C.47	C.55	C.56	C.67	C.69	C.94
Number of sequence	9	8	7	3	4	5	6

Table B2

Protein folds	A.1	C.1	C.2	C.3	C.47
Number of sequence	1	3	4	1	1

It can be seen from the above statistics that for 24 of the PPM IndelFR predictors out of the 29 predictors, there is no protein sequence that is common between their training sets and the test set for the proposed MSAIndelFR algorithm. It should be pointed out that for any of the predictors for the remaining 5 folds, the maximum overlap between the training set and the test set is less than 12.6%.

In the PREFAB 4.0 benchmark, we have selected 863 reference alignments that use 838 different protein sequences. The distribution of these protein sequences over the protein folds is given in Table C1. We have determined that only 6 of these 838 protein sequences were involved in training the PPM IndelFR predictors. The distribution of these 6 protein sequences over the protein folds is listed in Table C2.

Table C1

Protein folds	A.1	A.3	A.4	A.22	A.25	A.26	A35	A.39	A.45	B.6	B.18
Number of sequence	24	9	39	11	11	28	4	15	7	18	8
Protein folds	**B.29**	**B.34**	**B.35**	**B.36**	**B.40**	**B.42**	**B.47**	**B.50**	**B.55**	**B.60**	**B.82**
Number of sequence	17	10	4	5	33	14	15	13	22	12	8
Protein folds	**C.1**	**C.2**	**C.3**	**C.14**	**C.23**	**C.26**	**C.36**	**C.37**	**C.47**	**C.55**	**C.56**
Number of sequence	87	66	11	8	30	13	2	68	30	23	15
Protein folds	**C.61**	**C.68**	**C.69**	**C.94**	**C.95**						
Number of sequence	8	12	37	22	3						

Table C2

Protein folds	A.4	B.34	B.55	C.3	C.37	C.94
Number of sequence	1	1	1	1	1	1

It can be seen from the above statistics that for 32 of the PPM IndelFR predictors out of the 38 predictors, there is no protein sequence that is common between their training sets and the test set for the proposed MSAIndelFR algorithm. It should be pointed out that for any of the predictors for the remaining 6 folds, the maximum overlap between the training set and the test set is less than or equal to 10.0%.

In the SABRE (SABmark 1.65) benchmark, we have selected 79 reference alignments that use 542 different protein sequences. The distribution of these protein sequences over the protein folds is given in Table D1.

Table D1

Protein folds	A.1	A.4	A.45	A.133	B.6	B.34	B.35	B.36	B.40	B.42	B.47
Number of sequence	4	10	22	3	31	43	4	16	16	26	33

Protein folds	B.50	B.55	B.60	B.82	B.121	C.1	C.3	C.14	C.23	C.26	C.36
Number of sequence	11	19	9	11	21	60	21	7	41	9	17

Protein folds	C.55	C.56	C.61	C.67	C.68	C.69	C.95
Number of sequence	4	15	11	26	8	19	18

We have found that for none of the 29 PPM IndelFR predictors there is any protein sequence that is common between its training sets and the test set for the proposed MSAIndelFR algorithm.

In view of the above discussion, it can be concluded that the training set for any of the PPM IndelFR predictors is virtually different from the test set of the proposed MSAIndelFR algorithm on all the four benchmarks.
